# IL‐10 induces MC3T3‐E1 cells differentiation towards osteoblastic fate in murine model

**DOI:** 10.1111/jcmm.14832

**Published:** 2019-11-21

**Authors:** Yuan Xiong, Chenchen Yan, Lang Chen, Yori Endo, Yun Sun, Wu Zhou, Yiqiang Hu, Liangcong Hu, Dong Chen, Hang Xue, Bobin Mi, Guohui Liu

**Affiliations:** ^1^ Department of Orthopaedics Union Hospital Tongji Medical College Huazhong University of Science and Technology Wuhan China; ^2^ Division of Plastic Surgery Brigham and Women's Hospital Harvard Medical School Boston MA USA; ^3^ Department of Neurosurgery Union Hospital Tongji Medical College Huazhong University of Science and Technology Wuhan China

**Keywords:** Fracture, IGF1R, IL‐10, miRNA, mRNA, osteoblast

## Abstract

Interleukin‐10 (IL‐10) displays well‐documented anti‐inflammatory effects, but its effects on osteoblast differentiation have not been investigated. In this study, we found IL‐10 negatively regulates microRNA‐7025‐5p (miR‐7025‐5p), the down‐regulation of which enhances osteoblast differentiation. Furthermore, through luciferase reporter assays, we found evidence that insulin‐like growth factor 1 receptor (IGF1R) is a miR‐7025‐5p target gene that positively regulates osteoblast differentiation. In vivo studies indicated that the pre‐injection of IL‐10 leads to increased bone formation, while agomiR‐7025‐5p injection delays fracture healing. Taken together, these results indicate that IL‐10 induces osteoblast differentiation via regulation of the miR‐7025‐5p/IGF1R axis. IL‐10 therefore represents a promising therapeutic strategy to promote fracture healing.

## 1 INTRODUCTION

Fracture is very common in clinic, a rapid healing of fracture can help patient get early recovery, decrease the occurrence of certain related complications, as well as improving the quality of life. Osteoblast differentiation is vital during bone remodelling and fracture healing and is regulated by an array of biological factors.^1^, ^2^, ^3^ MC3T3‐E1 cells are precursors of osteoblasts, which are established from a C57BL/6 mouse calvaria and selected on the basis of high alkaline phosphatase (ALP) activity in the resting state.^4^, ^5^ In a previous study, it is reported that MC3T3‐E1 cells secrete collagen and express murine leukaemia inhibitory factor (mLIF) in RNA.^6^ However, the lack of knowledge on the mechanisms underlying MC3T3‐E1 differentiation continues to hinder the progress of MC3T3‐E1‐based therapies for bone regeneration. IL‐10 has profound and indispensable functional effects on infection, inflammation, tissue homeostasis, autoimmunity and cancer.^7^, ^8^, ^9^, ^10^, ^11^ In recent years, the IL‐10 family have been shown to regulate arthritis,^12^, ^13^ suggesting potential effects on osteoblast differentiation. Similarly, a recent study had reported that overexpression of IL‐10 could disrupt osteoclast differentiation via NF‐ĸB signalling.^13^ However, the underlying mechanism of IL‐10 in the regulation of osteogenic differentiation is still elusive. Herein, it is necessary to catch a better understanding of the osteoblastic functionality of IL‐10.

microRNAs (miRNAs) are short (20‐24 nt) non‐coding RNAs involved in the post‐transcriptional regulation of gene expression in multicellular organisms by affecting both mRNA stability and translation.^14^, ^15^ During recent decades, suppressive miRNAs have been shown to exert regulatory functional effects through targeting osteogenic genes involved in osteoblast growth, osteoclast‐mediated bone absorption processes and bone balance.^16^, ^17^, ^18^, ^19^ To investigate the association between miRNAs and osteogenic differentiation, we obtained data sets (http://www.ncbi.nlm.nih.gov/geo/query/acc.cgi?acc=GSE76197) related to bone formation from the Gene Expression Omnibus (GEO) (https://www.ncbi.nlm.nih.gov/geo/). We identified the down‐regulation of miR‐7025‐5p in six mice (three intact control samples and three post‐fracture samples) that implicated its regulatory role during osteoblast differentiation.

In this study, we investigated the role of IL‐10 in the modulation of osteoblast differentiation both in vitro and in vivo. We demonstrate that IL‐10 positively influences osteoblast differentiation through its ability to down‐regulate miR‐7025‐5p. The down‐regulation of miR‐7025‐5p could induce IGFIR mRNA expression and accelerate fracture healing. Taken together, these results demonstrate that IL‐10 promotes osteoblast differentiation through regulation of the miR‐7025‐5p/IGF1R axis.

## 2 MATERIALS AND METHODS

### 2.1 Animal fracture models

Male C57BL/6J mice (age 6 weeks) were purchased from the Center of Experimental Animal, Tongji Medical College, Huazhong University of Science and Technology. We used 10% chloral hydrate (0.3 mL/100 g bodyweight) for anaesthesia. The Institutional Animal Care and Use Committee at Tongji Medical College, Huazhong University of Science and Technology approved all animal studies. The mouse femoral fracture model was created surgically via longitudinal incision and blunt separation of the underlying muscles without removal of the periosteum. A diamond disk was used to cut the femur, producing a transverse osteotomy in the mid‐diaphysis region. Fractures were stabilized via a 23‐gauge intramedullary needle. After 14 days, 50% of the mice were sacrificed, and the calluses at the fracture location were harvested for subsequent analysis. On day 21 post‐operation, the remaining mice were sacrificed, and the calluses were harvested for subsequent analysis.

### 2.2 Imaging of small animals in vivo

All small animals were imaged at the central laboratory of Wuhan Union Hospital (Wuhan Union Hospital; http://www.whuh.com/). Animals were assessed on days 0, 4, 7, 10 and 14 following local direct injections of 100 µL of 200 µmol/L Cy3‐labelled agomiR‐7025‐5p (GenePharma). Mice were anaesthetized 15‐minutes post‐injection with 200 µL 1% chloral hydrate solution and imaged using an in vitro FX PRO imaging system (BRUKER) with a 1‐minute exposure time.

### 2.3 Radiographic images

On days 7, 14 and 21 post‐injury, fractured femurs were scanned using the FX PRO imaging system with a 10‐seconds exposure time.

### 2.4 Micro‐CT analysis

The fracture site was scanned using a BRUKER SkyScan 1276 scanner microCT system (BRUKER) to provide images at 2400 views, 5 frames/view, 37 kV and 121 mA. Three‐dimensional images were rendered and evaluated using CT‐Vox2.1 version (BRUKER Minimal Intensity Projection Software). Soft tissues were thoroughly cleaned. After scanning, calluses were preserved at −80°C for miRNA extraction and PCR and Western blot. Measurement parameters were as follows: bone volume (BV), total volume (TV) and BV/TV.

### 2.5 Cell culture and transfection

MC3T3‐E1 cells (CODE: 99072810, European Collection of Authenticated Cell Cultures) were maintained in α‐MEM media (HyClone, #SH30265.01B) containing 10% FBS (Gibco, #10099141) and 1% penicillin and streptomycin (HyClone, #SV30010). Transfection of agomiR‐7025‐5p, agomiR‐NC, antagomiR‐7025‐5p and antagomiR‐NC (GenePharma) at 200 μmol/L was performed with lipofectamine 3000 (Thermo Fisher Scientific, #L3000001) based on the manufacturers protocol. Lipofectamine 3000 was also used for miRNAs or siRNA oligo transfections. IGF1R siRNA (GenePharma) was transfected at 50 nmol/L.

### 2.6 Quantitative real‐time PCR

qRT‐PCR assays were performed as previously described.^20^ Briefly, total RNA was prepared using TRIzol reagent (Thermo Fisher Scientific, #L15596026). First‐strand cDNA was synthesized using the qPCR RT Master Mix (Toyobo). Relative gene expression levels of mRNA were calculated using the 2^−ΔΔCt^ method (Ct of GAPDH minus the Ct of the target genes) and the relative expression of miRNA was normalized against U6 and determined as the 2^−ΔΔCt^. Primer sequences are listed in Table 1.

**Table 1:** miRNAs and mRNA primer sequence

microRNAs or gene name	Primer sequence (5′ to 3′)
Mmu‐miR‐702‐5p‐Forward	ACACTCCAGCTGGGCGTGAGCTGAAGCTGGTG
Mmu‐miR‐7025‐5p‐Reverse	TGGTGTCGTGGAGTCG
U6‐Forward	CTCGCTTCGGCAGCACA
U6‐Reverse	AACGCTTCACGAATTTGCGT
Mmu‐IGF1R‐Forward	TGCCCTGATATGCTGTTTGA
Mmu‐IGF1R ‐ Reverse	GGCTTGTTCTCCTCGCTGT
Mmu‐ALP‐Forward	TGACTACCACTCGGGTGAACC
Mmu‐ALP‐Reverse	TGATATGCGATGTCCTTGCAG
Mmu‐COL1A1‐Forward	CTGACTGGAAGAGCGGAGAG
Mmu‐COL1A1‐Reverse	CGGCTGAGTAGGGAACACAC
Mmu‐OCN‐Forward	TTCTGCTCACTCTGCTGACCC
Mmu‐OCN‐Reverse	CTGATAGCTCGTCACAAGCAGG
Mmu‐Runx2‐Forward	CGCCACCACTCACTACCACAC
Mmu‐Runx2‐Reverse	TGGATTTAATAGCGTGCTGCC
Mmu‐GAPDH‐Forward	AGAGTGTTTCCTCGTCCCG
Mmu‐GAPDH‐Reverse	CCGTTGAATTTGCCGTGA

### 2.7 Western blot

Cell lysates were prepared using NETN buffer (20 mmol/L Tris HCl, pH 8.0, 100 mmol/L NaCl, 1 mmol/L EDTA and 0.5% Nonidet P‐40) and were resolved by SDS‐PAGE. Proteins were transferred to PVDF membranes and blocked in 5% skimmed milk at 4℃overnight. Membranes were probed with primary antibodies and labelled with HRP‐conjugated secondary antibodies (Aspen, #AS1058). Chemiluminescence detection systems (Canon, #LiDE110) were used to visualize protein bands. Antibodies were as follows: anti‐collagen I (1:500; Abcam, #ab34710), anti‐ALP (1:1000; Abcam, #ab95462), anti‐Osteocalcin (1:500; Abcam, #ab93876), anti‐RunX2 (1:500; Abcam, #ab23981), anti‐IGF1R (1:2000; CST) and anti‐GAPDH (1:10 000; Abcam, #ab37168).

### 2.8 Alkaline Phosphatase staining

A BCIP/NBT alkaline phosphatase (ALP) colour development kit (Beyotime, #C3206) was used based on provided directions to assess ALP staining. Briefly, after washing twice with PBS, cells were fixed with 10% formalin for 15 minutes. The BCIP/NBT liquid substrate was then applied to cells for 24 hours. Samples were prepared in the dark at room temperature. Colour changes were obtained under a charge‐coupled device (CCD) microscope, and the stained cell cultures were imaged using a scanner (EPSON V600). Samples were analysed in triplicate.

### 2.9 Alizarin red staining

MC3T3‐E1 cells were grown in osteogenic media supplemented with 100 nmol/L dexamethasone, 50 mmol/L ascorbic acid and 10 mmol/L b‐glycerophosphate (Cyagen, #HUXMA‐90021) in 6‐well plates to induce osteoblast mineralization. Cells were fixed in formalin (10%) at room temperature for 15 minutes, washed in 2 mL PBS and stained using 1 mL 0.5% alizarin red staining solution at room temperature for 15 minutes. Cells were rinsed with distilled water for 5 minutes with shaking, and red mineralized nodules were assessed via a CCD microscope and scanned (EPSON V600). All staining data were repeated three times.

### 2.10 Luciferase reporter assays

The putative miR‐7025‐5p recognition sites in the anti‐IGF1R 3ʹ UTR were predicted by TargetScan 7.2 (http://www.targetscan.org/vert_72/), which is showed in Figure 6A. Mouse IGF1R 3′UTR containing an miR‐7025‐5p binding sequence was amplified via qRT‐PCR from murine genomic DNA and subcloned into pGL3 (Promega, #E1741). Quick Change Site‐Directed Mutagenesis Kits (Stratagene, #210518) were used for mutagenesis. MC3T3‐E1 cells (2.5 × 10^5^ cells per well) were transiently transfected with 100 ng luciferase constructs and 10 ng pRL‐TK in 24‐well plates using lipofectamine 3000 (ThermoFisher Scientific, #L3000001), Promega, #E2241). Dual‐luciferase reporter assays (Promega, #E1910) were performed on a luminometer (Glomax, Promega). Firefly luciferase activity was normalized to Renilla luciferase.

### 2.11 Stimulation of IL‐10/agomiR‐7025‐5p in fracture models

Mice were injected locally at the fracture sites. A volume of 0.1 mL per injection was administered on days 1, 3 and 7 following fracture induction. IL‐10 (Cyagen, #MEILP‐1001) or agomiR‐7025‐5p (GenePharma) was directly injected in local sites. Following experimental completion, animals were euthanized, and bones and calluses were isolated. qRT‐PCR and Western blot analysis were performed.

### 2.12 Statistical analysis

Data are presented as the means ± SD, and GraphPad Prism 8.0 (GraphPad Software, Inc) was used for all analyses unless otherwise stated. A one‐way analysis of variance using a Tukey's post‐hoc test was applied to compare three or more groups. A two‐tailed Student's test was applied for data comparisons between the groups. *P* < .05 was deemed statistically significant.

## 3 RESULTS

### 3.1 IL‐10 accelerates fracture healing in vivo

**Figure 1:**
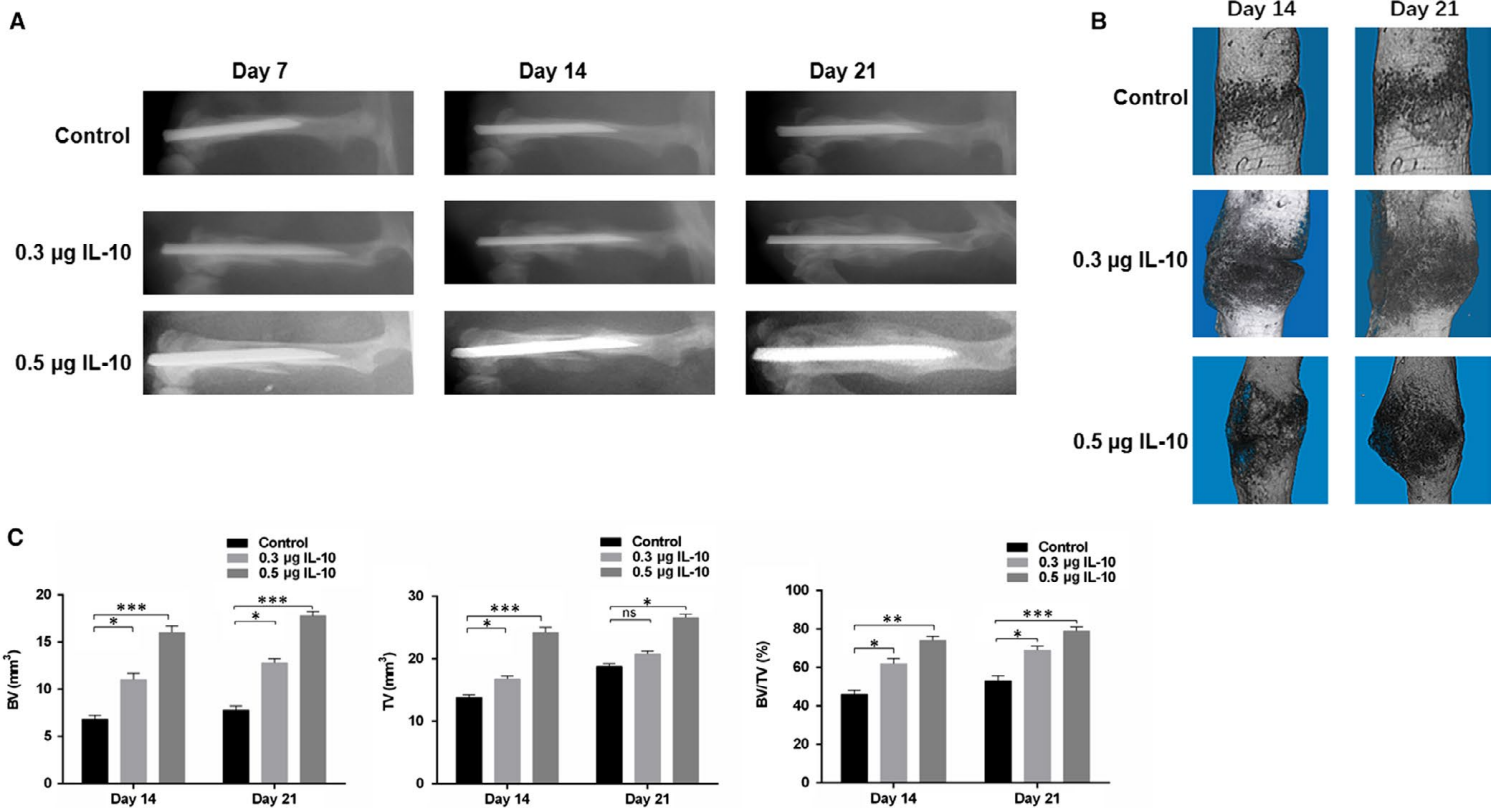
Local Administration of IL‐10 Enhances Fracture Healing in Mice. A, X‐rays images comparing fracture healing amongst control, 0.3 µg/kg IL‐10 and 0.5 µg/kg IL‐10 groups on days 7, 14 and 21 post‐injury. B, micro‐CT images comparing fracture healing amongst control, 0.3 µg/kg IL‐10 group and 0.5 µg/kg IL‐10 groups on days 14, and 21 post‐injury. C, BV, TV and BV/TV of the calluses on days 14 and 21 post‐operation were established via micro‐CT. n = 10 mice/group. Data are means ± SD of triplicate experiments. *P < .05, **P < .01, ***P < .001

To demonstrate the effects of IL‐10 on fracture healing, we administered phosphate‐buffered saline (PBS), 0.3 µg IL‐10 or 0.5 µg IL‐10 directly into the local fracture sites of model animals. Local injections were performed on days 1, 3 and 7 post‐injury, and X‐rays and micro‐CT (m‐CT) examinations were performed on days 14 and 21 post‐injury. Mice treated with 0.3 µg and 0.5 µg IL‐10 exhibited a greater callus volume and smaller fracture gap relative to control animals on days 14 and 21(Figure 1A,B). Furthermore, animals administered 0.3 µg and 0.5 µg IL‐10 showed greater total bone callus volumes relative to control animals on post‐fracture day 14, and the difference remained significant on post‐fracture day 21 (Figure 1C). These results indicate that IL‐10 promotes fracture healing in vivo.

### 3.2 IL‐10 promotes osteoblast differentiation in vitro

**Figure 2:**
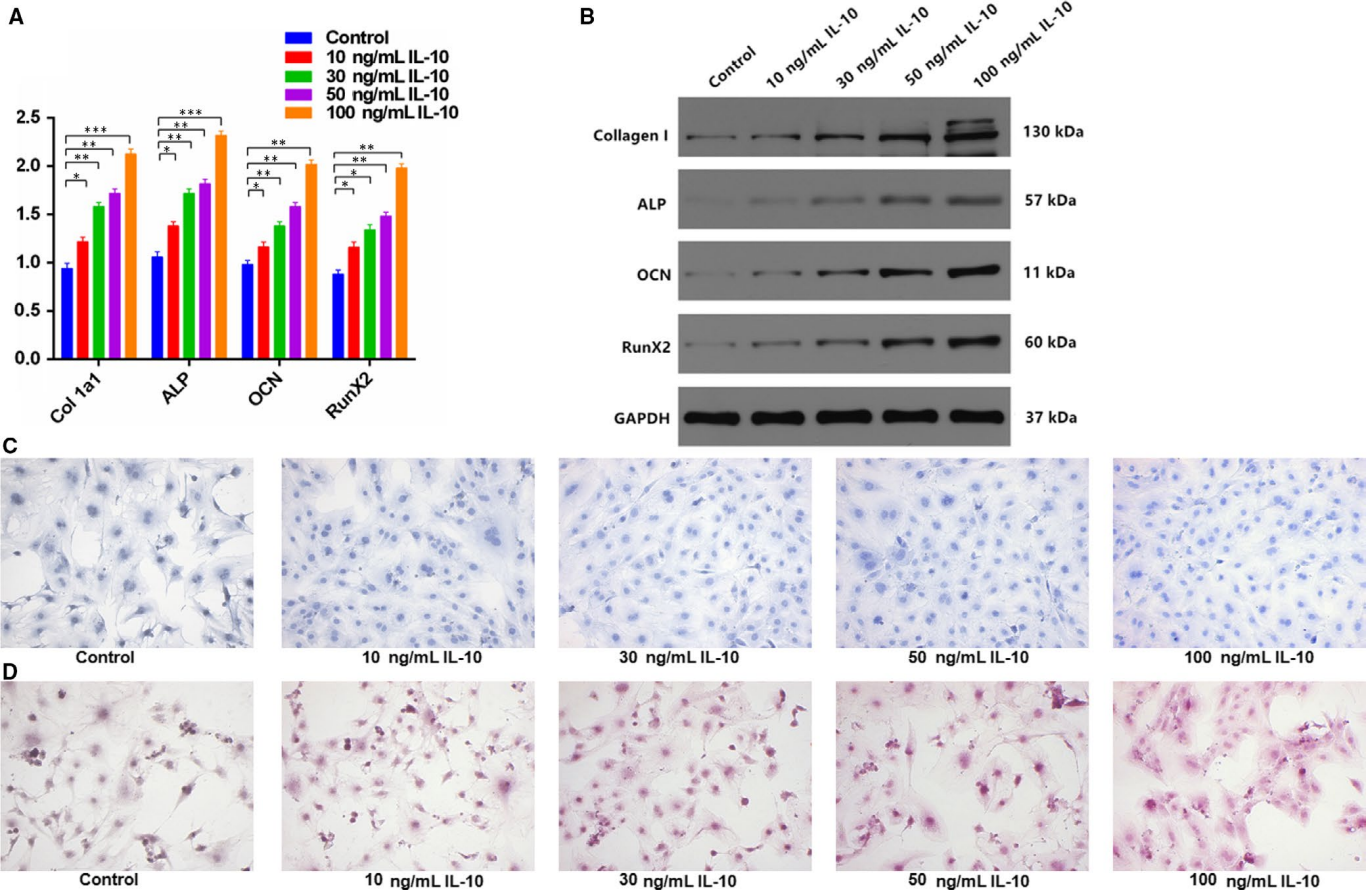
IL‐10 Promotes Osteoblast Differentiation in MC3T3‐E1 Cells. A, MC3T3‐E1 cells were divided into five groups based on IL‐10 treatments. Osteogenic markers Col 1a1, ALP, OCN and RunX2 in the five groups were measured by qRT‐PCR. B, Western blot of Col 1a1, ALP, OCN and RunX2 in (A)**.** C, ALP staining in MC3T3‐E1 cells transfected with various concentration of IL‐10 for 48 hours. Scale bar = 10 mm. D, Alizarin red‐mediated calcium staining in MC3T3‐E1 cells following transfection with various concentration of IL‐10 for 21 days. Scale bar = 10 mm. Data are means ± SD of triplicate experiments. *P < .05, **P < .01, ***P < .001

To explore the association between IL‐10 and osteoblast differentiation, MC3T3‐E1 cells were treated with various concentrations of IL‐10 (0, 10, 30, 50 and 100 ng/mL) for 24 hours, and osteoblastsogenesis alpha‐1 type I collagen (Col 1a1), alkaline phosphatase (ALP), osteocalcin (OCN) and runt‐related transcription factor 2 (RunX2) were determined via qRT‐PCR and Western blot analysis. As shown in Figure 2A,B, IL‐10 promoted osteoblast differentiation and the levels of osteogenic markers in the IL‐10‐treated groups showed a concentration‐dependent increase. The effects of IL‐10 on extracellular matrix mineralization in MC3T3‐E1 cells were investigated through the treatment of various concentrations of IL‐10 (0, 10, 30, 50 and 100 ng/mL) and after continuous culture, higher mineral deposition was observed in IL‐10‐treated groups relative to controls (Figure 2C,D). These results demonstrated that IL‐10 positively regulates osteoblastsogenesis and activates ALP activity and mineralization.

### 3.3 IL‐10 promotes osteoblast differentiation through the inhibition of miR‐7025‐5p

**Figure 3:**
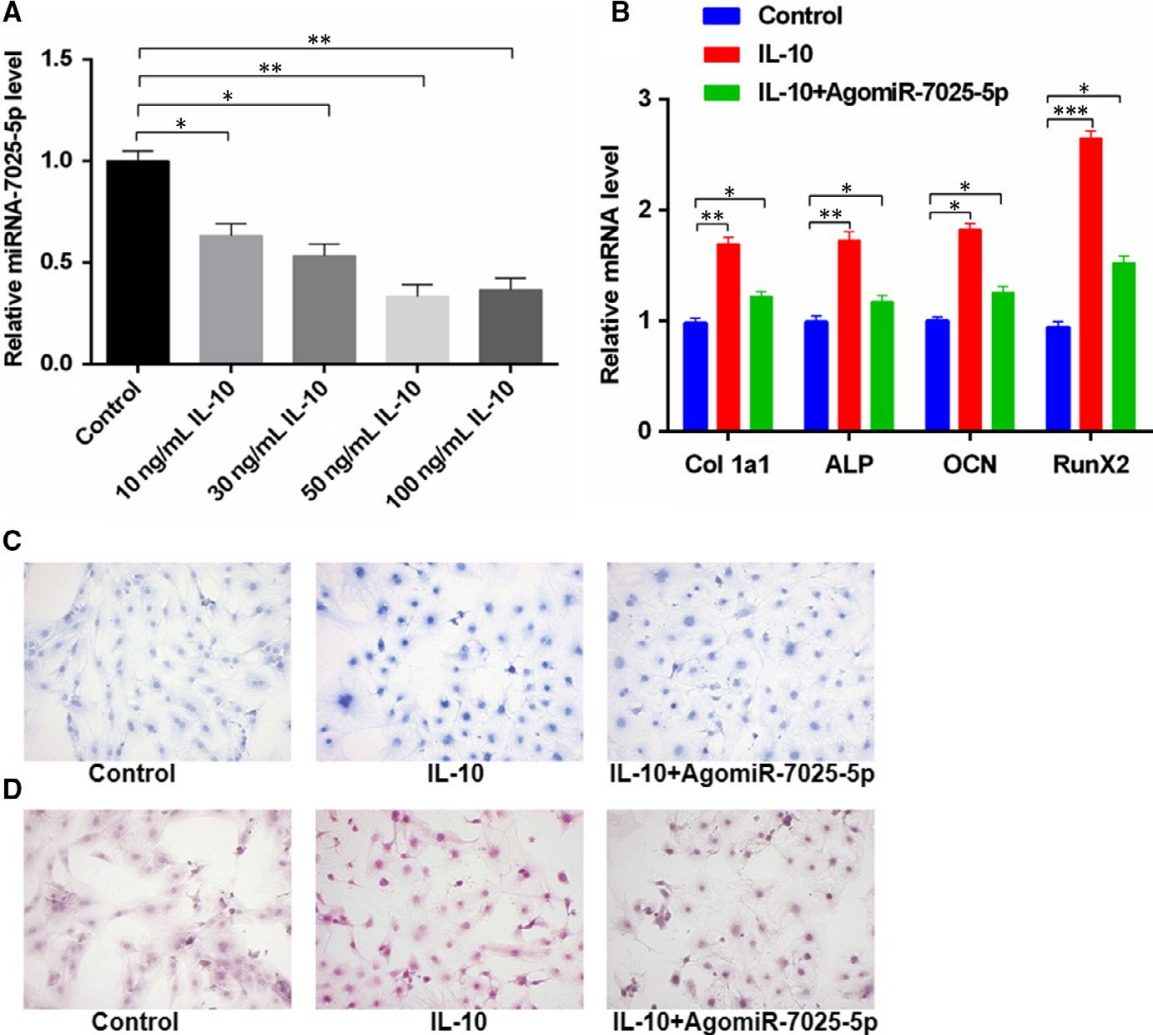
MiR‐7025‐5p is Down‐regulated and Osteogenic Differentiation Increases in IL‐10‐Treated Groups. A, MC3T3‐E1 cells were treated with various concentrations of IL‐10 (0, 10, 30, 50 and 100 ng/mL) for 24 hours, and miR‐7025‐5p levels in the IL‐10‐treated groups were significantly lower than the control group. B, MC3T3‐E1 cells were treated with lipofectamine 3000 and IL‐10, or IL‐10 **+ **agomiR‐7025‐5p. A significant increase in the expression of osteogenic markers in the IL‐10 group relative to the other groups was observed by qRT‐PCR analysis. C, ALP staining from (B)**.** D, Alizarin red‐mediated calcium staining in (B). Scale bar = 10 mm. Data are means ± SD of triplicate experiments. *P < .05, **P < .01, ***P < .001

We next compared the levels of miR‐7025‐5p in response to IL‐10. MC3T3‐E1 cells were treated with IL‐10 (0, 10, 30, 50 and 100 ng/mL) for 24 hours, and miR‐7025‐5p expression was assessed via qRT‐PCR analysis. As shown in Figure 3A, miR‐7025‐5p levels in the IL‐10‐treated groups showed a concentration‐dependent decrease, suggesting that IL‐10 suppresses miR‐7025‐5p expression. To investigate the relationship between IL‐10 and miR‐7025‐5p during osteoblast differentiation, MC3T3‐E1 cells were treated a control transfection reagent, IL‐10 or IL‐10 **+ **agomiR‐7025‐5p for 48 hours, and the expression of collagen, ALP, OCN and RunX2 genes was assessed by PCR and Western blot. We observed a significant increase in the expression of all osteogenic markers in IL‐10‐treated groups, which was inhibited through the expression of the miR‐7025‐5P mimic (Figure 3B). Moreover, when transfected cells were assessed for extracellular matrix mineralization after continuous culturing, higher mineral deposition was obvious in the IL‐10 group relative to other groups (Figure 3C,D). Collectively, these data suggest that IL‐10 positively regulates osteoblastsogenesis through the inhibition of miR‐7025‐5p.

### 3.4 Negative effects of miR‐7025‐5p on fracture healing in vivo

**Figure 4:**
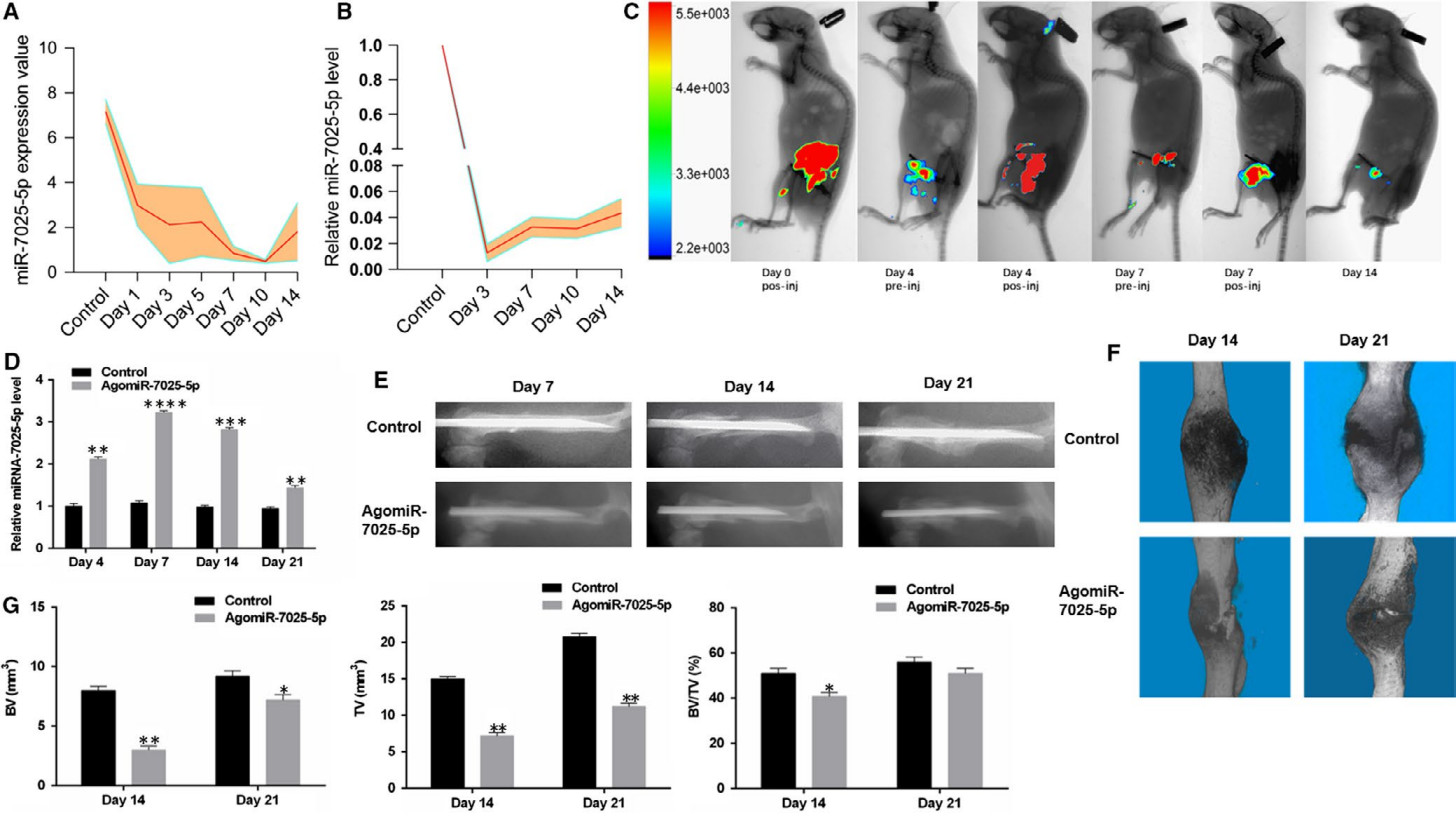
Local Administration of AgomiR‐7025‐5p Inhibits Healing in Mice. A, Levels of miR‐7025‐5p decreased in the gene chips during the early period of fracture healing. B, Levels of miR‐7025‐5p decrease in the fracture models during the early stages of fracture healing. C, Imaging of small animals in vivo to assess the effects of agomiR‐7025‐5p at the fracture sites. D, High levels of miR‐7025‐5p were found in the calluses of agomiR‐7025‐5p animals on days 4 and 7 by qRT‐PCR analysis. E, Mice treated with agomiR‐7025‐5p exhibited a longer healing time relative to control animals in X‐rays. F, Mice treated with agomiR‐7025‐5p exhibited a smaller callus volume and enlarged fracture gap relative to control animals in 3D m‐CT. G, Reduced total bone callus volume in agomiR‐7025‐5p animals relative to control animals on days 14 and 21 post‐fracture by m‐CT data analysis. Data are the mean ± SD of triplicate experiments. *P < .05, **P < .01, ***P < .001

To explore the relationship between miR‐7025‐5p and bone formation, we calculated the relative levels of miR‐7025‐5p in mice fracture gene chips (Figure 4A). Calluses from the fracture sites in mouse models were also collected for the assessment of miR‐7025‐5p levels (Figure 4B). We found that miR‐7025‐5p expression markedly decreased during the early stages of fracture healing. To further investigate the role of miR‐7025‐5p in vivo*,* mice fracture models were randomly divided into two groups (control and receiving agomiR‐7025‐5p). In the agomiR‐7025‐5p group, all animals received local injection of fluorescent miR‐7025‐5p into the fracture sites on days 0, 4 and 7 post‐injury, and animals were imaged to assess the levels of miR‐7025‐5p in the fracture sites (Figure 4C). High levels of miR‐7025‐5p were found in the calluses of agomiR‐7025‐5p animals on days 4, 7, 14 and 21 by qRT‐PCR (Figure 4D). Moreover, when X‐rays and CT examinations were performed to compare the speed and quality of fracture healing, mice treated with agomiR‐7025‐5p exhibited a smaller callus volume and larger fracture gap relative to control animals (Figure 4E,F). Additionally, in the agomiR‐7025‐5p animals, there was a smaller total bone callus volume relative to control animals on post‐fracture day 14, and the difference remained significant between agomiR‐7025‐5p and control animals on post‐fracture day 21 (Figure 4G).These results indicate that miR‐7025‐5p acts as a negative regulator of fracture healing.

### 3.5 MiR‐7025‐5p inhibits osteoblast differentiation and matrix mineralization in vitro

**Figure 5:**
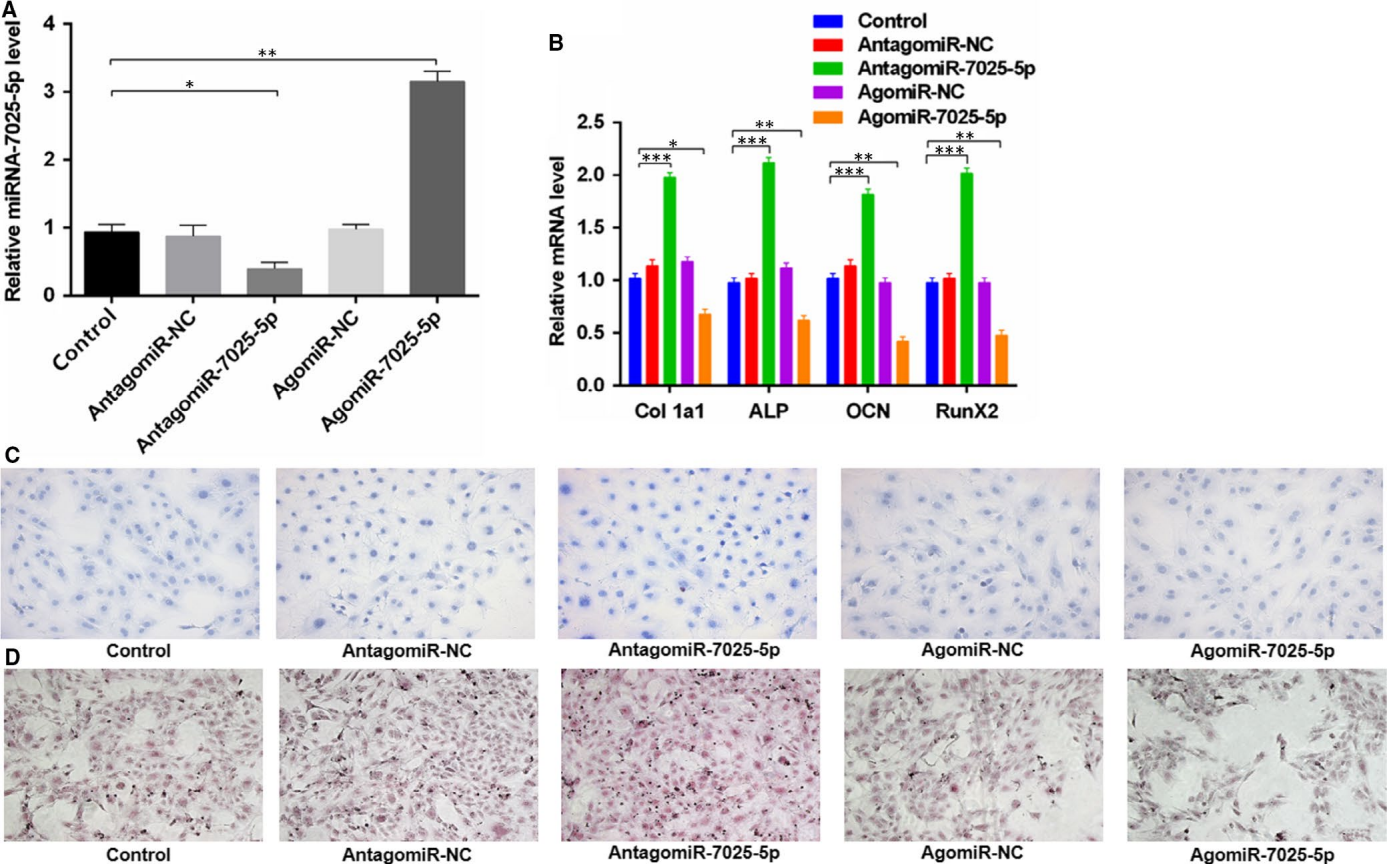
MiR‐7025‐5p‐Negative Regulates Osteoblast Differentiation in vitro. A, MC3T3‐E1 cells were treated with lipofectamine3000 alone, agomiR‐NC, agomiR‐7025‐5p, antagomiR‐NC or antagomiR‐7025‐5p lipofectamine. miR‐7025‐5p was up‐regulated in cells treated with agomiR‐7025‐5p assessed through qRT‐PCR analysis. B, qRT‐PCR analysis of osteogenic markers Col 1a1, ALP, OCN and RunX2 in (A). C, ALP staining results from (A). D, Alizarin red‐mediated calcium staining in (A). Scale bar = 10mm. Data are means ± SD of triplicate experiments. *P < .05, **P < .01, ***P < .001

We next investigated the effects of miR‐7025‐5p on osteoblast differentiation by treating MC3T3‐E1 cells with transfection reagent, antagomiR‐negative control (antagomiR‐NC), antagomiR‐7025‐5p, agomiR‐negative control (agomiR‐NC) or agomiR‐7025‐5p. We found that miR‐7025‐5p was up‐regulated in cells treated with agomiR‐7025‐5p (Figure 5A). The in vitro effects of miR‐7025‐5p were then evaluated through the investigation of osteoblast activity and the expression of the bone formation related genes Col 1a1 or collagen I, ALP, OCN and RunX2. After 48 hours transfection, qRT‐PCR analysis revealed a significant increase in the expression of the bone formation markers in the antagomiR‐7025‐5p group relative to the other groups (Figure 5B). When the influence of miR‐7025‐5p on extracellular matrix mineralization was assessed, higher mineral deposition was observed in the antagomiR‐7025‐5p group, particularly when compared to agomiR‐7025‐5p groups (Figure 5C,D). Taken together, these results reveal that miR‐7025‐5p negatively regulates osteoblastsogenesis and thereby suppressing ALP activity and mineralization.

### 3.6 MiR‐7025‐5p directly targets IGF1R

**Figure 6:**
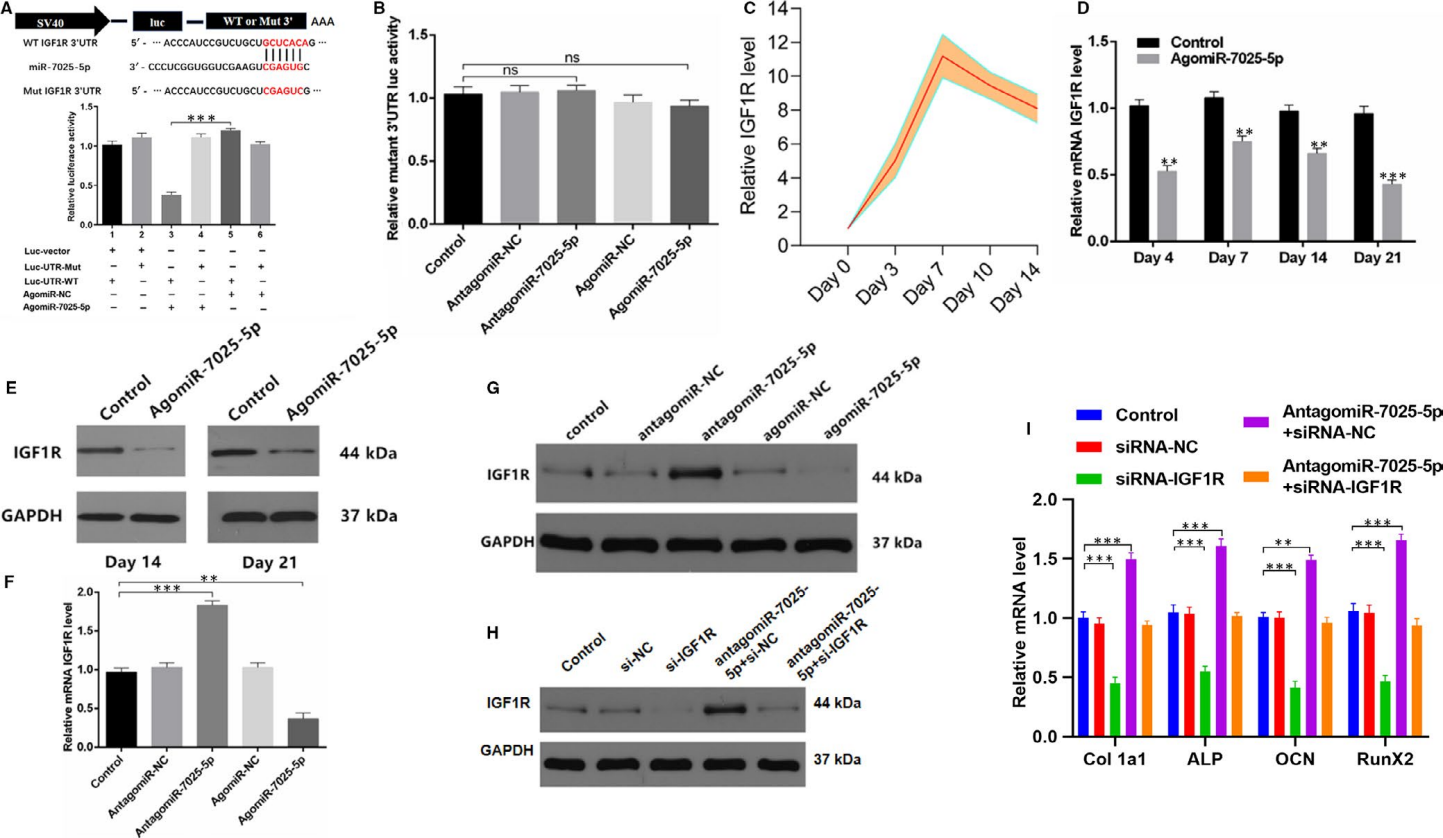
IGF1R is a Target Gene for MiR‐7025‐5p. A, AgomiR‐7025‐5p decreases WT IGF1R 3′ UTR reporter activity. B, No significant difference in the activity of the mutated 3′ UTR IGF1R reporter among the groups. C, Elevated levels of IGF1R in the calluses of fracture animals detected during the early stages of fracture healing (days 0, 3, 7, 10 and 14). D, Reduced mRNA levels of IGF1R in serum samples detected by PCR analysis in the agomiR‐7025‐5p group (days 4, 7, 14 and 21). E, Western blots of IGF1R in callus samples between the two groups on days 14 and 21. F, PCR analysis of IGF1R mRNA levels in the transfected groups (control group, agomiR‐NC group, agomiR‐7025‐5p group, antagomiR‐NC group and antagomiR‐7025‐5p group). G, Western blots of IGF1R mRNA levels in (F). H, Western blots of IGF1R mRNA levels in MC3T3‐E1 cells transfected with mock, siRNA‐NC, siRNA‐IGF1R, antagomiR‐7025‐5p + siRNA‐NC or antagomiR‐7025‐5p + siRNA‐IGF1R. (I) qRT‐PCR analysis of collagen I, ALP, OCN and RunX2 in (H). Data are the means ± SD of triplicate experiments. *P < .05, **P < .01, ***P < .001

To explore whether miR‐7025‐5p targets IGF1R directly, we expressed either wild‐type (WT) IGF1R 3′ UTR or mutant‐type (Mut) IGF1R 3′ UTR constructs fused to luciferase reporters and assessed their expression in response to antagomiR‐7025‐5p or miR‐7025‐5p. Using these constructs, we found that agomiR‐7025‐5p substantially attenuated WT IGF1R 3′ UTR reporter activity (Figure 6A), but failed to influence the activity of the mutated 3′ UTR IGF1R reporter (Figure 6B). IGF1R was continuously detected for 14 days during the early stages of fracture and increased during these stages (Figure 6C). In vivo, calluses were collected from the two groups (control and agomiR‐7025‐5p) on days 4, 7, 14 and 21 to detect the levels of IGF1R by PCR analysis. Lower levels of IGF1R mRNA were detected in the agomiR‐7025‐5p group compared with the control group on each day (Figure 6D). Furthermore, calluses of the fracture sites were harvested from animals in the two groups on days 14 and 21, and Western blot analysis showed that levels of IGF1R were significant lower in agomiR‐7025‐5p compared with control groups (Figure 6E). In vitro, MC3T3‐E1 cells were transfected with transfection reagent (control), antagomiR‐negative control (agomiR‐NC), antagomiR‐7025‐5p, agomiR‐negative control (antagomiR‐NC), or agomiR‐7025‐5p and IGF1R mRNA levels were measured by qRT‐PCR (Figure 6F) and Western blot (Figure 6G). We observed higher relative IGF1R mRNA levels in the antagomiR‐7025‐5p group compared with the other groups. In addition, to investigate whether osteoblast differentiation is IGF1R‐dependent, we assessed whether antagomiR‐7025‐5p can rescue the negative effects of siRNA‐IGF1R on osteogenic differentiation. Western blot and qRT‐PCR analysis revealed that antagomiR‐7025‐5p could restore IGF1R dependent gene expression during osteogenesis, including collagen I, ALP, OCN and RunX2 (Figure 6H‐I). These results demonstrate that miR‐7025‐5p directly targets the IGF1R 3′ UTR during osteoblast differentiation.

## 4 DISCUSSION

**Figure 7:**
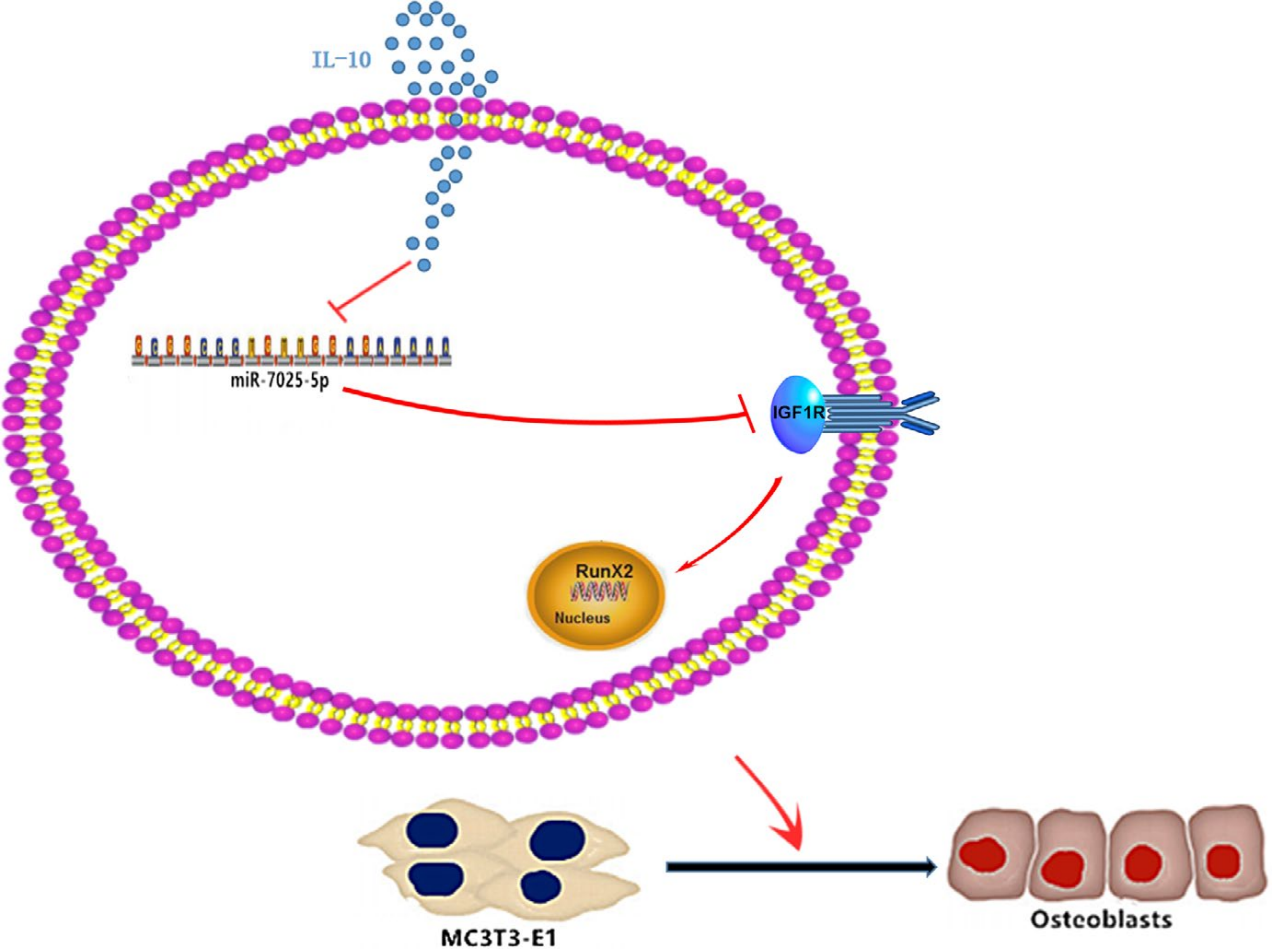
Schematic of the Effects of IL‐10 on Osteoblast Differentiation. IL‐10 down‐regulates the levels of miR‐7025‐5p, thereby inducing the expression of IGF1R, subsequently stimulating osteogenic expression, increasing bone formation

In this study, we evaluated the role of IL‐10 during the process of osteogenic differentiation. Our results suggested that IL‐10 positively regulates osteogenic processes both in vitro and in vivo through regulation of the miR‐7025‐5p/IGF1R axis (Figure 7). In addition, we evaluated the expression of miR‐7025‐5p and IGF1R in serum and femur bone calluses of fracture mice and identified alterations in the miR‐7025‐5p/IGF1R axis in both samples.

IL‐10 targets both innate and adaptive immune responses and exerts immunosuppressive functions to reduce tissue damage caused by excessive and uncontrolled inflammatory effector responses, particularly during the resolution phase of infection and inflammation, to maintain homeostasis to gut microbes.^21^, ^22^, ^23^ Subsequent studies focused on the role of IL‐10 during osteoblast differentiation. Osteoblast is responsible for bone formation,^24^, ^25^, ^26^ and MC3T3‐E1 cells are precursors of osteoblasts. Since IL‐10 regulates arthritis development,^12^, ^13^ we speculated that IL‐10 is involved in the osteogenic capacity of MC3T3‐E1 cells. We found that manipulating the concentration of IL‐10 significantly alters the osteogenic capacity of MC3T3‐E1 cells both in vitro and in vivo. These results, for the first time, emphasize the critical role of IL‐10 not only in immunosuppressive functions but during skeletal osteogenic differentiation.

The question remained as to how IL‐10 regulates osteogenic differentiation. Since the suppression of miRNA exerts regulatory functions in progressive osteogenic pathways, osteoblast growth, osteoclast‐mediated bone absorption processes and bone balance via targeting osteogenic genes,^16^, ^17^, ^18^, ^19^ we searched for differentially expressed miRNAs between control and post‐fracture samples and selected miR‐7025‐5p from the GEO data set. Subsequently, the regulatory role of IL‐10 on miR‐7025‐5p expression was demonstrated in vitro. To explore the role of miR‐7025‐5p during osteoblast differentiation, we screened for miR‐7025‐5p interacting partners in MC3T3‐E1 cells. We sought to explore miR‐7025‐5p downstream targets via screening miRDB data sets, target scan data sets and GEO data sets for potential targets. We can identify three candidate genes of interest –TRIAP1, IGF1R and SHANK2. A previous study reported that expression of prostaglandin F2α could stimulate DNA synthesis and proliferation by up‐regulation of IGF1R in MC3T3‐E1 cells.^27^ And following a literature review, we found IGF1R to be the most associated with osteogenic differentiation of these three genes.^20^, ^28^ We therefore next assessed whether miR‐7025‐5p directly targets IGF1R, using either wild‐type (WT) IGF1R 3′UTR or mutated (Mut) IGF1R 3′UTR constructs fused to luciferase reporters. And through luciferase assays, we identified IGF1R as a downstream target. IGF1R is receptor tyrosine kinase that mediates the actions of insulin‐like growth factor 1 (IGF1). IGF1 binds to IGF1R with high affinity whilst IGF2 and insulin (INS) bind with lower affinity. Activated IGF1R is involved in cell growth and survival and influences an array of physiological and pathological conditions.^29^, ^30^, ^31^ For example, IGF1R inhibition is an escape mechanism in Ewing sarcoma and IGF1R inhibitors are candidate therapies for this disease.^32^ In this study, we found that IGF1R interacts with miR‐7025‐5p and that the down‐regulation of miR‐7025‐5p increases the IGF1R mRNA levels to promote osteogenic processes. Moreover, we found the levels of IGF1R and miR‐7025‐5p remain stable during osteogenic differentiation, but the expression of the IGF1R protein increases during fracture healing, whilst the levels of miR‐7025‐5p decline. These results suggest that the miR‐7025‐5p/IGF1R axis forms a positive feedback cycle to promote the osteogenic differentiation of ME3T3‐E1 cells and decreases in miR‐7025‐5p‐mediated IGF1R degradation positively regulates the osteogenic capacity of MC3T3‐E1 cells. This adds to our current knowledge of the osteogenic process and the molecular mechanisms underlying MC3T3‐E1 osteogenic differentiation.

In recent decades, the regulatory factors of IL‐10 osteogenic processes have been intensely studied,^33^, ^34^, ^35^ However, previous studies have focused on anti‐inflammatory and immunosuppressive effects, and its role in osteogenesis has been less well studied, hampering the development of IL‐10‐based therapies for bone defects in the clinic. Our findings indicate that IL‐10 may be a prospective choice for bone repair therapies. We also discovered that IGF1R expression decreased in the calluses of mouse fracture samples with injection of agomiR‐7025‐5p. Previous studies demonstrated that abnormal IGF1R expression plays an important role in the pathogenesis of acute lymphoblastic leukaemia, adrenocortical carcinoma, colorectal cancer and Ewing sarcoma.^30^, ^36^, ^37^, ^38^, ^39^, ^40^ Thus, our findings indicate that the miR‐7025‐5p/IGF1R axis may play an important role in bone metabolism processes including osteoblast differentiation. Further studies on the miR‐7025‐5p/IGF1R axis may provide novel insights into understanding the pathogenesis of bone metabolism disorders, revealing new therapeutic targets.

Some limitations of this study remain. Firstly, we did not perform clinical studies to confirm our findings. In addition, to further explore the role of IL‐10 during osteogenesis, transgenic mice with specific IL‐10 receptor knockdowns in osteoblast lineages are required for validation.

In summary, we demonstrate that IL‐10 positively regulates fracture healing through regulation of the miR‐7025‐5p/IGF1R axis. IL‐10 therefore represents a potential therapeutic strategy to promote fracture healing in the clinic.

## CONFLICT OF INTEREST

The authors declare no conflict of interest.

## AUTHOR CONTRIBUTIONS

BM, HX and GL conceived and designed the study; YX, CY and LC supervised the study; YX, WZ and YS performed experiments; WZ, HX, YH and LH analysed the data; DC, FD and CY provided advice and technical assistance; and YX wrote the manuscript. YX was supported by the China Scholarship Council for 18 months study at the Harvard Medical School. All authors approved the final manuscript.

## Data Availability

The data that support the findings of this study are available from the corresponding author upon reasonable request.
