# Corrigendum

**DOI:** 10.1111/jcmm.15734

**Published:** 2021-01-15

In Xiong et al,^1^ the published article contains errors in Figure 1B and Figure 4F. The correct figures are shown below. The authors confirm all results and conclusions of this article remain unchanged.

**FIGURE 1:**
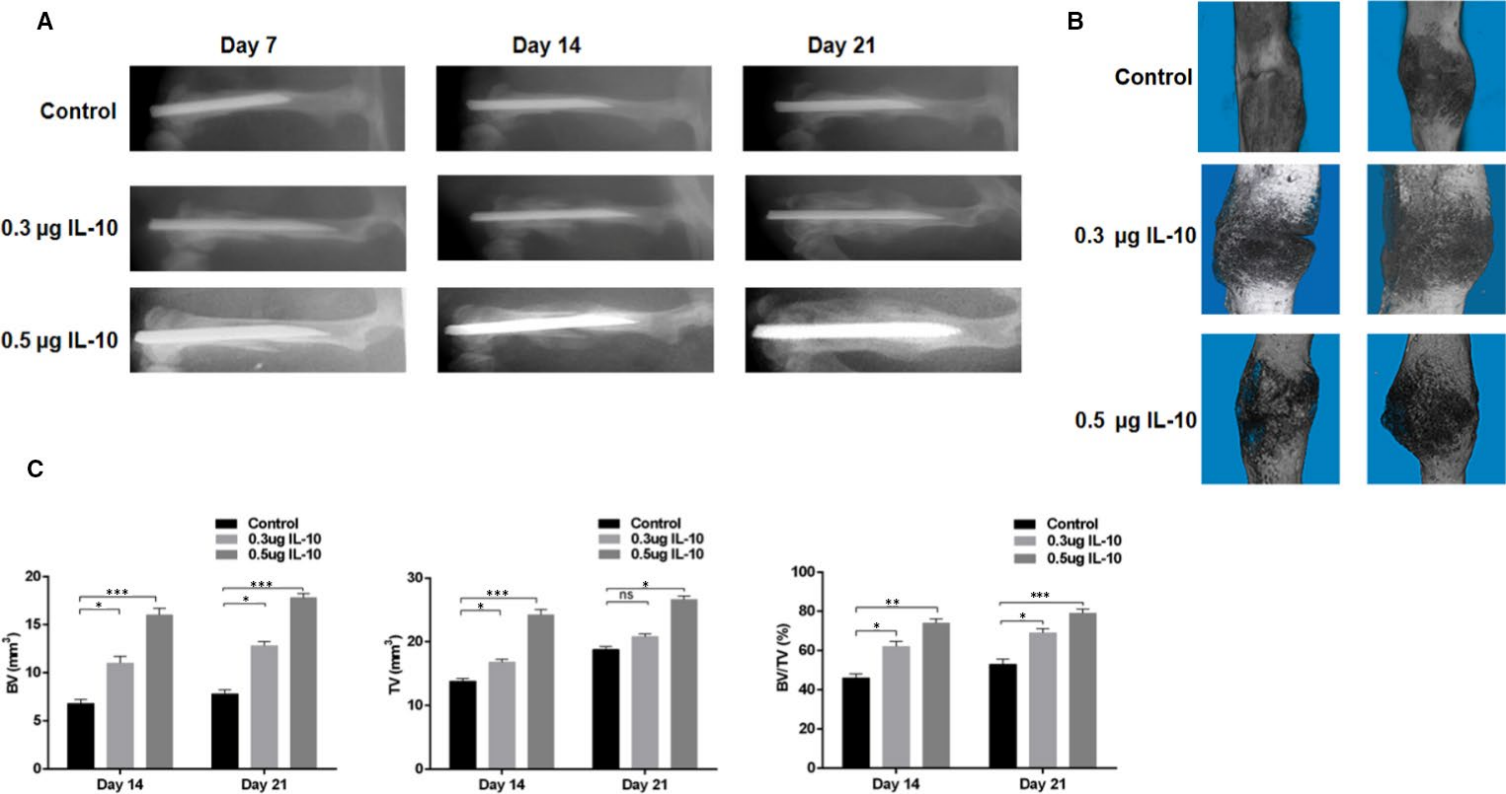
Local Administration of IL‐10 Enhances Fracture Healing in Mice. A, X‐ray images comparing fracture healing amongst control, 0.3 μg/kg IL‐10 and 0.5 μg/kg IL‐10 groups on days 7, 14 and 21 post‐injury. B, micro‐CT images comparing fracture healing amongst control, 0.3 μg/kg IL‐10 and 0.5 μg/kg IL‐10 groups on days 14 and 21 post‐injury. C, BV, TV and BV/TV of the calluses on days 14 and 21 post‐operation were established via micro‐CT. n = 10 mice/group. Data are means ± SD of triplicate experiments. *P < .05, **P < .01, ***P < .001

**FIGURE 4:**
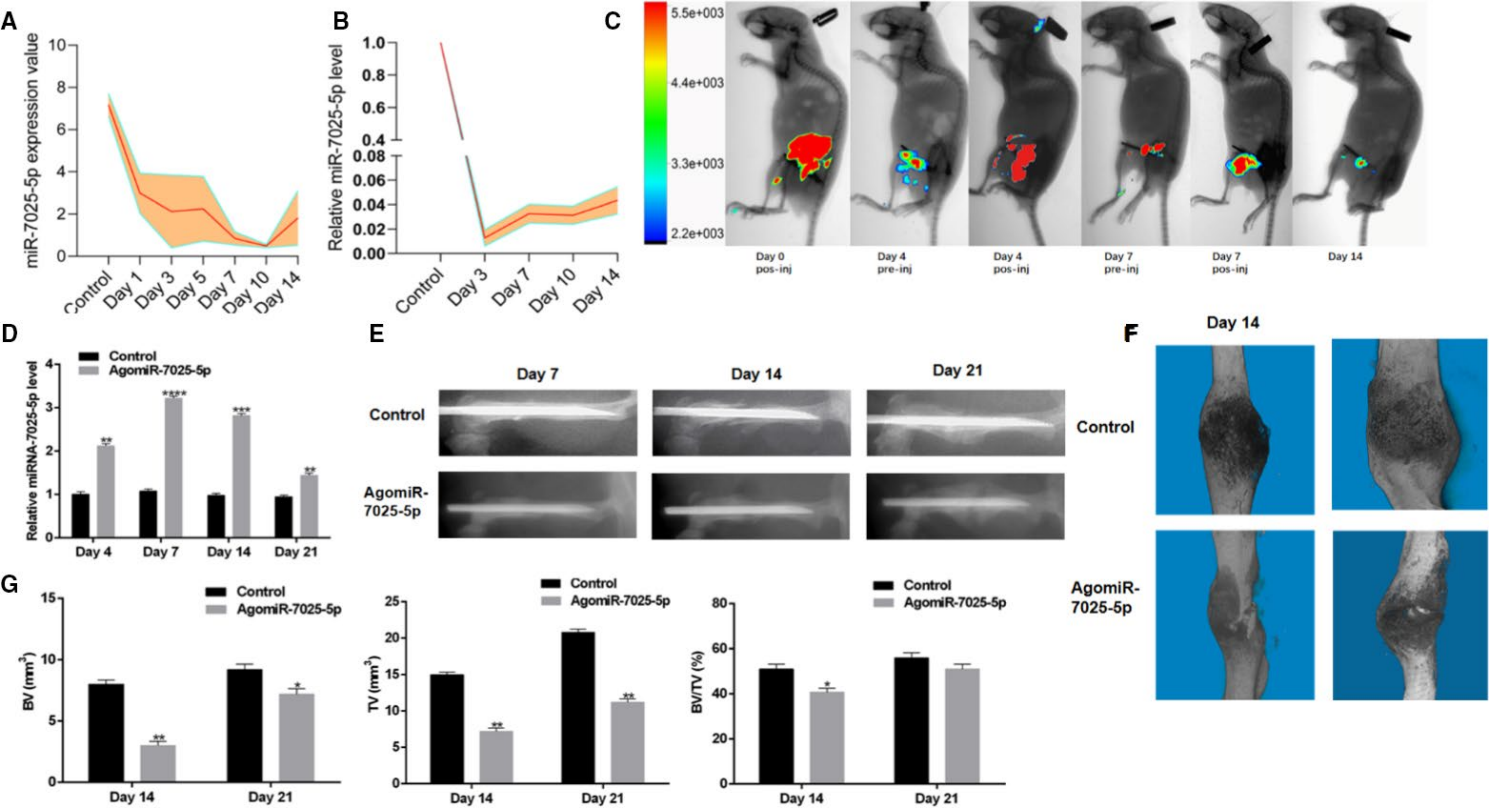
Local Administration of AgomiR‐7025‐5p Inhibits Healing in Mice. A, Levels of miR‐7025‐5p decreased in the gene chips during the early period of fracture healing. B, Levels of miR‐7025‐5p decrease in the fracture models during the early stages of fracture healing. C, Imaging of small animals in vivo to assess the effects of agomiR‐7025‐5p at the fracture sites. D, High levels of miR‐7025‐5p were found in the calluses of agomiR‐7025‐5p animals on days 4 and 7 by qRT‐PCR analysis. E, Mice treated with agomiR‐7025‐5p exhibited a longer healing time relative to control animals in X‐rays. F, Mice treated with agomiR‐7025‐5p exhibited a smaller callus volume and enlarged fracture gap relative to control animals in 3D m‐CT. G, Reduced total bone callus volume in agomiR‐7025‐5p animals relative to control animals on days 14 and 21 post‐fracture by m‐CT data analysis. Data are the mean ± SD of triplicate experiments. *P < .05, **P < .01, ***P < .001
